# A Distance-Aware Replica Adaptive Data Gathering Protocol for Delay Tolerant Mobile Sensor Networks

**DOI:** 10.3390/s110404104

**Published:** 2011-04-06

**Authors:** Yong Feng, Haigang Gong, Mingyu Fan, Ming Liu, Xiaomin Wang

**Affiliations:** School of Computer Science and Engineering, University of Electronic Science and Technology of China, Chengdu 610054, China; E-Mails: fybraver@163.com (Y.F.); ff98@163.com (M.F.); csmliu@uestc.edu.cn (M.L.); xiaomin.wang@126.com (X.W.)

**Keywords:** delay tolerant mobile sensor networks, wireless sensor networks, data gathering scheme, replica adaptive, delivery probability

## Abstract

In Delay Tolerant Mobile Sensor Networks (DTMSNs) that have the inherent features of intermitted connectivity and frequently changing network topology it is reasonable to utilize multi-replica schemes to improve the data gathering performance. However, most existing multi-replica approaches inject a large amount of message copies into the network to increase the probability of message delivery, which may drain each mobile node’s limited battery supply faster and result in too much contention for the restricted resources of the DTMSN, so a proper data gathering scheme needs a trade off between the number of replica messages and network performance. In this paper, we propose a new data gathering protocol called DRADG (for Distance-aware Replica Adaptive Data Gathering protocol), which economizes network resource consumption through making use of a self-adapting algorithm to cut down the number of redundant replicas of messages, and achieves a good network performance by leveraging the delivery probabilities of the mobile sensors as main routing metrics. Simulation results have shown that the proposed DRADG protocol achieves comparable or higher message delivery ratios at the cost of the much lower transmission overhead than several current DTMSN data gathering schemes.

## 1. Introduction

Delay Tolerance Mobile Sensor Networks (DTMSNs) [1–5] have several unique characteristics such as sparse network density, short range radio and sensor node mobility, energy limits and so on, which result in intermittent connectivity and frequently changing network topology. Obviously, it is difficult to form well connected end-to-end paths for mobile sensor nodes to transmit data messages to the sink nodes in a DTMSN. Mobile nodes usually use probabilistic delivery schemes, that is, in the process of data delivery, a node and its neighbors form the temporary route for delivering the data in a carry-and-forward scheme. Since the probabilistic delivery cannot guarantee the data delivery performance, it is reasonable to employ multi-replica transmission schemes, which means generating multiple replicas for every data message and spreading these copies to different nodes, in order to enhance the successful delivery ratio and reduce delivery delays.

Though multi-replica transmission schemes are generally efficient for improving data gathering performance, these schemes have to expend much more network resources, such as bandwidth, node energy and buffer space *etc.* compared to single-replica transmission schemes. For a DTMSN whose resources are usually limited, too many duplicate messages will dramatically increase traffic overhead, cause a large amount of wireless collisions, increase overall delays, and rapidly drain mobile nodes’ limited battery energy. Therefore, an efficient data gathering scheme tailored for DTMSNs needs to maximize data delivery ratios while minimizing communication overhead (*i.e*., the number of data message replicas).

In recent years, a number of multi-replica routing protocols [6–10] have been proposed to enable data delivery in such challenging environments as DTMSNs. These multi-replica protocols can be classified into two categories based on the number of replicas generated and injected to the network: flooding-based and quota-based. Flooding-based protocols send a replica of each message to as many nodes as possible, whereas quota-based protocols intentionally limit the number of replicas.

A majority of existing multi-replica protocols, such as Epidemic [6], MaxProp [7], PREP [8], RAPID [9] and *etc.* are flooding-based. Though these protocols can effectively reduce the number of duplicate messages compared with the basic flooding algorithm, they usually fail to work well in DTMSNs. The main reason is that their high demands on network resources that are typically unavailable in such challenging environments as DTMSNs. Different from flooding-based routings, quota-based protocols such as Spray-and-Wait [10] and Spray-and-Focus [11] use fixed numbers of duplicate messages, which effectively limits network resource expansion and thus make them attain better performance than flooding-based routing schemes.

However, quota-based protocols still have performance problem due to the fact that each message is delivered and transferred in the same and fixed amount of replicas, which would cause unwanted results as follows: (a) when a node is very close to the sink node, the number of duplicates exceeds the need, thereby causing redundancy and when the node is far enough from the sink node, the number of duplicates may not meet the performance needs; (b) the quota-based protocols do not take the difference of node delivery capability into account, and simply consider that every node has the same capability to deliver data to the sink nodes. However, in practice there are obvious differences between the delivery capabilities of nodes.

This paper proposes a novel data gathering scheme, called Distance-aware Replica Adaptive Data Gathering protocol (DRADG), which is tailored for DTMSNs. By elaborately adapting the number of duplicates for every data message and computing the delivery probability so that data messages are forwarded to nodes with higher delivery capability, DRADG can achieve high network performance with low transmission overhead cost. Through intensive simulation, we evaluate the data gathering performances of the proposed protocol and several existing schemes, and the results show that DRADG protocol achieves comparable or higher message delivery ratios at the cost of the lower transmission overhead than the existing schemes.

The rest of the paper is organized as follows: we review the related work in Section 2 and identify the problems in the existing works. In Section 3, we present the DRADG protocol in detail. The simulation is carried out, and the performance is evaluated in Section 4. Finally, we conclude this work in Section 5.

## 2. Related Work

A DTMSN is fundamentally an opportunistic network in which the topology frequently changes and communication links exist with certain probability, therefore, replication-based efficient data delivery scheme has its rationale. In epidemic routing [6], a sensor will duplicate its data messages to any neighbor in communication range only if the neighbor does not carry a copy of the data message. Obviously, network resources, such as bandwidth, buffer, battery, supply and so on, have a great influence on the performance of epidemic protocols. Given enough network resources, epidemic routing can achieve very high data delivery rate at the cost of huge traffic overhead. However, the bandwidth, buffer and nodes’ energy are limited in DTMSNs, thus the epidemic protocol is not applicable for them.

Spray-and-Wait [10] is a representative quota-based scheme, in which every message is delivered and forwarded with a same upper bound on the amount of replicas. Spray-and-Wait routing includes two phases: (a) spray phase, where data message replicas are generated and injected into the network; (b) wait phase, where data messages with single-quota will not be forwarded until a direct encounter with the respective destinations occurs. Spray-and-Focus [11] is very similar to Spray-and-Wait, and the difference between the two protocols is that Spray-and-Focus allows forwarding of data messages with single-quota in order to improve performance. Although both protocols succeed in limiting some of the traffic overhead compared with flooding-based protocols, their network performances still suffer.

Wang and Wu [12] presented a replication-based efficient data delivery called RED, which consists of two components for data delivery and message management. First, data delivery uses a history-based method like ZebraNet to calculate the delivery probabilities of sensor nodes. Second, the message management algorithm decides the optimal erasure coding parameters based on sensor’s current delivery probability to improve the data delivery ratio. However, as indicated in [3], the optimization of erasure coding parameters is usually inaccurate, especially when the source is very far away from the sinks. In [13], Wang and Wu [12] also proposed a FAD protocol to increase the data delivery ratio in DTMSNs. Besides using the same delivery probability calculation method as RED, FAD further discusses how to constrain the number of data replications in the sensor network by using a fault tolerance value associated to each data message. However, that protocol still has a quite high transmission overhead.

Recently, several new routing protocols such as MaxProp [7], PREP [8], RAPID [9] and OPF [14] have been proposed to achieve the desired performance. OPF assumes that all nodes have full routing information, that is, the mean inter-meeting times between all pairs of nodes. Though the authors discuss how to release the assumption from full routing information to partial routing information, the assumption is still strong. MaxProp, PREP and RAPID are new examples of flooding-based protocols. They all attempt to mitigate the inherent resource burden from the basic flooding protocol. However, their traffic overheads are still considerable high, and thus they cannot be considered ideal schemes for DTMSNs.

## 3. Distance-Aware Replica Adaptive Data Delivery Scheme

As is described above, on the one hand, DRADG dynamically decides the replica number for every data message through sensing the distance between the node, which generates the message, and the sink nodes. On the other, DRADG computes the delivery probability of each mobile node, and forwards data messages to nodes with higher delivery probability. Therefore, DRADG protocol can reach high data gathering performance. In this section, we will describe the proposed DRADG protocol in detail.

### 3.1. Network Model

**Figure 1.**
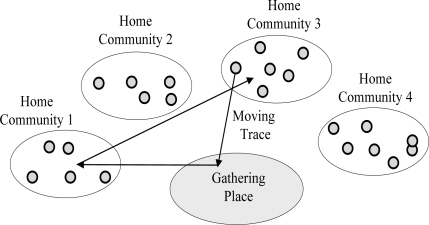
Illustration of community-based mobility model.

Without loss of generality, we consider a network that consists of *N* sensors randomly deployed in a square area *A*. Each sensor and gathering point has the same radius *r*. And we assume the mobile sensor network has the following character:

- All sensors’ movement accord with the Community-based Mobility model [15], which can be described as follows: in the community-based mobility model, the whole network area *A* consists of a gathering place (e.g., in reality it can be a market for people, or a feeding ground for animals), and some communities. Each node has one home community that it is more likely to visit than other places, and for each community there are a number of nodes that have it as home community. Each node selects a destination and moves to it at a selected speed *v* (*v_min_*, *v_max_*), and then repeats the process. The destination is selected such that if a node is a home community, there is a high probability that it will go to the gathering place, of course it is also possible for the node to go to other places); and if it is away from home, it is very likely that the node will return home. The mobility of every mobile node follows the process described above and is mutually independent each other, as shown in Figure 1.
- The sink node is located at the gathering place. When the sink node is immobile, we assume that its location is known to all sensor nodes.
- Through either GPS (Global Positioning System) or other GPS-less technique such as those described in [16–18], each sensor node can compute its physical position.

### 3.2. Message Replica Number Calculation

To solve the performance problem that results from the fixed replica number in quota-based protocols, DRADG dynamically decides the replica number of each data message based on the distance between the sensor which generates the message and the sink node. We assume that each message head includes a field of integer type that keeps its replica ticket. The value of ticket denotes the upper bound of a message replica number.

Take node *i*, denoted as *n_i_*, for an example. When *n_i_* generates a new message *M* and just has an appropriate neighbor node for the next hop, it sets the ticket of M according to the following Equation 1:

(1) $${\mathrm{ticket}}_{M}=\left\lceil k\times T_{\text{max}}\times \sqrt{\frac{d_{i}}{D_{\text{max}}}}\right\rceil$$

where *k* ∈ (0,1) is scaling constant; *d_i_* is the current distance between *n_i_* and the sink node; *D_max_* is the longest distance between sensor nodes and the sink node in the whole network. Clearly, *D_max_* can be computed by the size of network and the location of the sink node. *T_max_* is the maximum value of ticket, that is, for any one message its ticket is an integer between 1 and *T_max_*. From Equation 1, it can be found that the ticket value increases with the distance of between *n_i_* and the sink node. By this way, we effectively avoid both the data message redundancy when sensors and the sink node are close to each other and the poor performance when they are far from each other.

### 3.3. Node Delivery Probability Calculation

In DRADG, each node calculates its delivery probability in accordance to its frequency of meeting with the sink node (*i.e.*, the frequency that the node moves into the communication range of the sink node and can directly communicate with it), and forwards data messages to nodes with high delivery probability in order to further enhance the data gathering performance. The delivery probability of a node indicates the possibility that the node can directly deliver data messages to the sink node. Let *P_i_* denote the delivery probability of *n_i_*. Due to the node mobility feature, the delivery probability varies with the change of the node’s activities, so each node needs to calculate the delivery probability periodically and broadcast the value to its neighbors through hello messages.

In the real world, the activities of mobile objects (e.g., people, vehicles or animals) usually show remarkable regularity and predictability [19], so we can predict the meeting probability of a mobile node within a future period of time based on the historic meeting records of the node. As to any node, e.g., *n_i_*, it maintains a timer with a timeout interval of *τ*, records its meeting times with the sink node in the latest interval of *τ*, denoted as *Num_i_*. Then the meeting frequency of *n_i_* in the most recent interval of *τ*, denoted as *freq_i_*, can be computed by Equation 2 as follows:

(2) $${\mathrm{freq}}_{i}=\{\begin{array}{ll} \frac{{\mathrm{Num}}_{i}}{{\mathrm{Num}}_{\mathrm{TH}}}, & {\mathrm{Num}}_{i}<{\mathrm{Num}}_{\mathrm{TH}}\\ 1, & {\mathrm{Num}}_{i}\geq {\mathrm{Num}}_{\mathrm{TH}} \end{array}$$

where *Num_TH_* is the threshold value that should be defined based on the application. Therefore, for *n_i_*, its delivery probability *P_i_* can be calculated as follows:

(3) $$P_{i}=\alpha \times {\mathrm{freq}}_{i}+(1-\alpha )\times P_{i}$$

In Equation 3, the exponentially weighted moving average places an emphasis proportional to *α* on the most recent interval *τ*.

### 3.4. Data Delivery Algorithm

By receiving hello messages, each node maintains a dynamic neighbor list in which it can acquire the delivery probability of every neighbor within its communication range. Take *n_i_* for example, let it have a data message *M* need to delivery, and its neighbor set be ∑ = {Ψz | 1 ≤ z ≤ Z}, where Z is the neighbor number of *n_i_*. Firstly *n_i_* looks for the neighbor with a highest delivery probability, denoted as *n_m_*, in set ∑.

**Figure 2.**
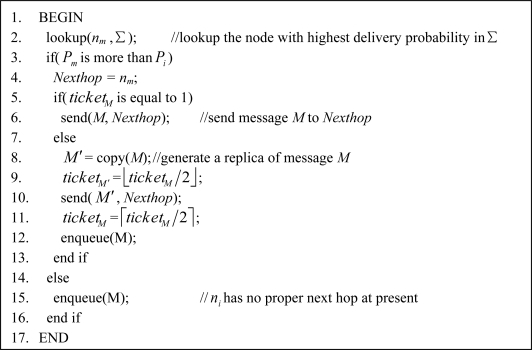
Pseudo-code of the data delivery algorithm.

If the delivery probability of *n_m_* is greater than that of *n_i_*, then *n_m_* is just regarded as the next hop; or else, there is no proper next hop at present, and the routing algorithm ends. Secondly, *n_i_* checks whether the ticket of *M*, denoted as *ticket_M_*, is equal to 1. If so, then *n_i_* directly transmits *M* to *n_m_*, routing decision completes. Otherwise, *i.e.*, *ticket_M_* ≥ 2, *n_i_* generates a replica *M*′ for message *M*, and set *ticket_M_*_′_ as ⌊*ticket_M_*/2⌋. Node *i* forwards *M*′ to *n_m_*. At last, *n_i_* updates *ticket_M_* as ⌈*ticket_M_*/2⌉ and then puts *M* into its routing queue. Detailed pseudo-code is shown in Figure 2.

### 3.5. Queue Management

Due to the limited buffer size of each node in a DTMSN, the buffer queue management would greatly influence the transmission performance. In DRADG, the ticket indicates how many replicas are allowed to be propagated, and the survival time shows how long a certain message has existed in the network. Therefore the ticket and survival time together denote the importance of a message, and the queue management is just based on the two parameters.

Messages are sorted in the routing queue based on a decreasing order of their tickets. For those with the same ticket value, they are further sorted according to an increasing order of survival time. Thus messages with larger tickets and shorter survival time are closer to the top of the queue, and can be transmitted with higher priorities. Moreover, messages will be dropped in the following two occasions: (a) when a message arrives and the queue is full, it is compared with that message at the end of the queue, and the one with less ticket is dropped among them. If the ticket values of the two messages are equal, then the one with a longer survival time is dropped; (b) whenever one message’s survival time is longer than the delay tolerance of the network, it is dropped to avoid unnecessary resource consumption.

## 4. Simulation

In this section, we perform the DRADG, FAD, Spray-and-Wait and epidemic routing protocols in NS-2.34, and compare the performances of the four protocols from the following points of view: data delivery ratio, data delivery delay, network traffic overhead, and network life. In addition, we also analyze the impacts of different experimental parameters on the protocols.

We assume the data generation of each sensor follows a Poisson process with an average arrival interval from 10 s to 100 s. Each sensor broadcasts a hello message to all its neighbors every 1.0 s, which is essential for mutual collaboration among sensors. The whole network area is divided into 3 × 3 subareas: eight communities, and one gathering place. Without loss of generality, the subarea at the left bottom is appointed as the gathering place, and the sink node is deployed at the place. The simulation parameters and their default values are summarized in Table 1.

### 4.1. Impact of Message Generation Rate

**Figure 3.**
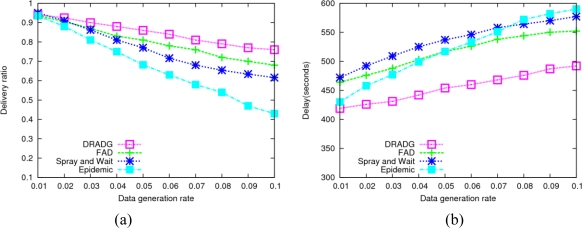
Impact of message generation ratio. **(a)** Average delivery ratio; **(b)** Average delay.

In this section, we vary the data generation rate in order to evaluate the performance of the four protocols under different transmission loads. As the date generation rate varies from 0.01 to 0.1 message/s, the performance of these protocols is as shown in Figure 3.

From Figure 3(a) we can see that all the four protocols achieve very high performance and their data delivery rates are close when the data generation rate is very low. However, as the data message generation rate goes up, the performance of the epidemic protocol decreases dramatically. This is due to MAC layer collision and rapid exhaustion of the limited network resources resulting from forwarding a tremendous amount of copies in epidemic routing. By using the fixed message replica number, which effectively limits the transmission overhead, Spray-and-Wait shows better performance than epidemic routing. In addition, we find that FAD outperforms Spray-and-Wait and the epidemic protocol as to the data delivery ratio. As the transmission load increases, its performance descends gradually since it is generating very many copies in this protocol. In general, DRADG achieves better performances than FAD, Spray-and-Wait and epidemic protocols, though its performance slowly decreases as the data generation ratio increases. The reason is that DRADG has less resource demands and can deal with high transmission loads by efficiently reducing the number of redundant data message replicas.

As shown in Figure 3(b), the average delivery delays of all protocols go up when the data generation rate increases. Obviously, the delivery delay of epidemic routing has the fastest speed increase among the four protocols. Because data messages with single-quota are not forwarded until a direct encounter with the respective destinations, Spray-and-Wait routing has higher delivery delay compared with the other three protocols in most cases. It can clearly be seen that our proposed DRADG routing has the shortest delivery delay among all protocols, because it efficiently cut down communication overhead as well as properly chooses next hop based on nodes’ delivery probabilities.

### 4.2. Impact of Buffer Size

**Figure 4.**
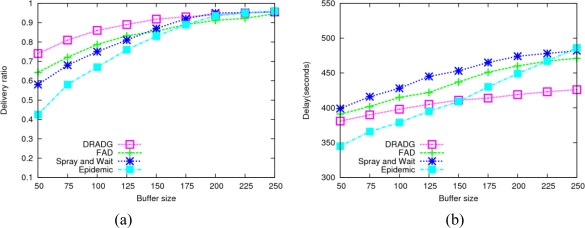
Impact of buffer size. **(a)** Average delivery ratio; **(b)** Average delay.

To evaluate the impact of buffer space on the performance, we vary the buffer size from 50 to 250 messages and get the delivery ratios and average delay of four protocols (see Figure 4). From Figure 4(a), we find that the data delivery ratio of all evaluated protocols increases as the buffer size increases, which shows the node buffer size has a remarkable influence on the performance of the multiple copy delivery protocols. Epidemic protocol is more sensitive to the buffer size compared to the others because it generates many redundant messages so that needs much more buffer space. Due to the fact that DRADG generates much less message replicas than the epidemic, FAD and Spray-and-Wait protocols, it needs less amount of buffer space. Therefore, DRADG outperforms the other three protocols most of the time. In Figure 4(b), it can be seen that the average delivery delays of all four protocols show an incremental trend when the node buffer size increases. The reason is that larger node buffer size leads to more data message exchange between nodes, so the chance of network congestion also increases.

### 4.3. Impact of Node Density

**Figure 5.**
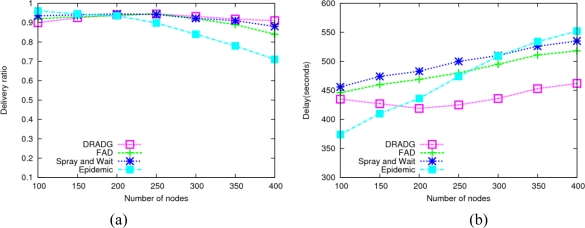
Impact of node density. **(a)** Average delivery ratio; **(b)** Average delay.

The connectivity of DTMSN is closely related to the density of sensor nodes. The following experiments show the network performance of the four protocols with different sensor node densities. As shown in Figure 5(a), the epidemic protocol almost achieves the upper bound of the data delivery ratio when the node density is very low, since low node density means low transmission load and a small amount of wireless collisions. As the node density increases, the number of message copies increases dramatically in epidemic routing, which results in an increasing number of collisions and the reduction of the data delivery ratio. FAD shows a slightly better data delivery ratio than DRADG when the node density is very low. This is due to poor connectivity resulting from very low node density, which influences the performance of DRADG. With the increment of node density, the connectivity of the network is enhanced, and thus the performance of DRADG improves rapidly. When the node number reaches 300, DRADG outperforms the other three protocols. As far as the performance of FAD and Spray-and-Wait are concerned, when the node density is high, both of the protocols will generate large numbers of message copies, which expends the limited resources of bandwidth and buffer quickly and results in the decrease of their performance.

In Figure 5(b), we can find that the delivery delay of DRADG reduces as the node density is increasing in the initial stage, because its performance is improved as the network connectivity is enhanced, but the delay of DRADG begins to go up due to the increasing communication overhead when the node number is more than 250. The delivery delays of the other three protocols show sustained trends with the increase of the node density. The reason is that the three protocols above have very high demand for network resources, and an increase of the node density aggravates the situation. Of course, epidemic routing has the fastest increase speed of the delivery delay among all four protocols.

### 4.4. Data Traffic Overhead

**Figure 6.**
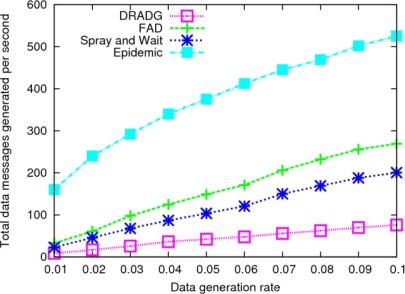
The number of data messages generated per second.

To evaluate the traffic overhead of the protocols, we use the number of data messages generated per second, which is a summation of individual message-hops [20]. For example, if a data message is forwarded five hops, the message overhead is counted as five message-hops. From Figure 6, we can see the number of messages generated by each of the four protocols increases as the data generation rate increases. However, the increase trends are very different from each other. The overhead of the epidemic protocol increases fastest among all protocols, and the traffic overhead of FAD is obviously higher than that of DRADG and Spray-and-Wait, which means FAD generates too many messages copies though it achieves high data delivery ratio. Due to its effective reduction of the number of message copies and proper selection of next hop nodes, we can clearly find that the traffic overhead of DRADG is the lowest in the four protocols.

### 4.5. Network Life

**Figure 7.**
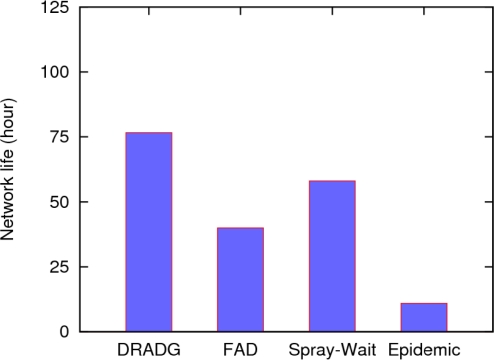
The network life under scenario 1.

**Figure 8.**
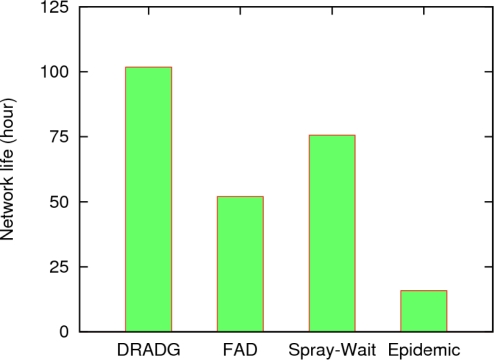
The network life under scenario 2.

The network life is an important assessment criterion of a protocol from the aspect of total energy consumption. The experiments show the network lifetime of the four protocols, and the results are described in Figures 7 and 8. We assume that the energy of the sink node is unlimited, and the initial energy of each sensor is 10 J. The energy needed in each transmission and receiving action is as specified in [21]. We consider the mobile sensor network dead according to the two scenarios as follows. Scenario 1: the network dies when any one of all sensor nodes depletes its energy; scenario 2: the network life ends when over a half of all sensor nodes deplete their energy.

From Figure 7, we can see that DRADG protocol achieves the longest network lifetime, since it makes use of a self-adapting algorithm to cut down the redundant replicas of messages, *i.e.*, reduce the consumption of nodes’ energy, and thus much energy can be saved. Since sending and receiving too many messages copies expends too much energy, the network lifetime of the epidemic protocol is the shortest among the four protocols. Moreover, we also see that Spray-and-Wait has much longer network life than FAD. The reason is that, Spray-and-Wait adopts fixed message copy number transmission scheme, thus it can efficiently limit communication overhead. Therefore, the total energy consumption of DRADG is much less than the Spray-and-Wait, FAD and epidemic protocols, which demonstrates the advantage of our proposed protocol in the aspect of economizing energy.

Figure 8 shows the network life of the four protocols under scenario 2. We can find that the network life values of all four protocols under scenario 2 go up significantly compared with the corresponding values in scenario 1. However, the size relationships of the network life values among these protocols remain unchanged. It can be clearly seen that the network life of DRADG protocol is still longest in all schemes and the network life of Spray-and-Wait is much longer than that of the FAD and epidemic protocols. To achieve a high data delivery ratio, FAD routing needs to generate and transfer a mass of data message copies, so its energy consumption is also very high. Similarly, the epidemic scheme has the shortest network life among the four protocols because of its enormous demand for the network resources. In a word, the above discussions, under both scenario 1 and 2, show that our proposed DRADG scheme clearly outperforms the Spray-and-Wait, FAD and epidemic protocols from the point of view of energy savings.

## 5. Conclusions

Data gathering schemes that can efficiently and effectively delivery data through intermittently connected networks are of critical importance to DTMSNs. In this paper, we propose a new data gathering scheme, called Distance-aware Replica Adaptive Data Gathering protocol (DRADG), which can provides better or comparable data gathering performance at the cost of much lower traffic overhead than current protocols. Extensive simulations have carried out for performance evaluation. The experimental results show that our proposed DRADG protocol not only achieves higher or comparable performance than several existing schemes in terms of data delivery ratio and transmission overhead, but also significantly outperforms them in the aspect of network traffic overhead.

## Figures and Tables

**Table 1.** Simulation parameters.

**Parameter**	**Default value**
Network size (m^2^)	1,500 × 1,500
Number of sensor node	200
Transmission radii *r* (m)	50
Speed of sensor node v (m/s)	1∼10
Maximum buffer size of sensor (message)	200
Data message size (bytes)	100
Control message size (bytes)	25
Message generation ratio (message/s)	0.01
Maximum delay tolerant value (s)	1,800
Position of sink node (m)	(300, 300)
Timer expiration value *τ* (s)	180
*Num_TH_*.	2
*α* value	0.8
*T_max_* value	10
